# The effect of QiangGuYin on osteoporosis through the AKT/mTOR/autophagy signaling pathway mediated by CKIP-1

**DOI:** 10.18632/aging.203848

**Published:** 2022-01-24

**Authors:** Yifeng Yuan, Jiangang Sun, Hang Zhou, Shen Wang, Caijian He, Tianpeng Chen, Mouhao Fang, Shaohua Li, Shifa Kang, Xiaosheng Huang, Binbin Tang, Bocheng Liang, Yingdelong Mao, Jianyou Li, Xiaolin Shi, Kang Liu

**Affiliations:** 1 The Second Clinical Medical College, Zhejiang Chinese Medical University, Hangzhou, China; 2 Department of Osteology, The Second Affiliated Hospital of Zhejiang Chinese Medical University, Hangzhou, China; 3 Department of Orthopedics, Huzhou Central Hospital, Huzhou, China

**Keywords:** osteoporosis, CKIP-1, AKT/mTOR signaling pathway, apoptosis, QiangGuYin

## Abstract

Osteoporosis is a systemic bone disease characterized by decreased bone mass and deterioration of bone microstructure, which leads to increased bone fragility and increased risk of fractures. Casein kinase 2 interacting protein 1 (CKIP-1, also known as PLEKHO1) is involved in the biological process of bone formation, differentiation and apoptosis, and is a negative regulator of bone formation. QiangGuYin (QGY) is a famous TCM formula that has been widely used in China for the clinical treatment of postmenopausal osteoporosis for decades, but the effect in regulating CKIP-1 on osteoporosis is not fully understood. This study aimed to explore the potential mechanism of CKIP-1 participating in autophagy in bone cells through the AKT/mTOR signaling pathway and the regulatory effect of QGY. The results *in vivo* showed that QGY treatment can significantly improve the bone quality of osteoporotic rats, down-regulate the expression of CKIP-1, LC3II/I and RANKL, and up-regulated the expression of p62, p-AKT/AKT, p-mTOR/mTOR, RUNX2 and OPG. It is worth noting that the results *in vitro* confirmed that CKIP-1 interacts with AKT. By up-regulating the expression of Atg5 and down-regulating the p62, the level of LC3 (autophagosome) is increased, and the cells osteogenesis and differentiation are inhibited. QGY inhibits the combination of CKIP-1 and AKT in osteoblasts, activates the AKT/mTOR signaling pathway, inhibits autophagy, and promotes cell differentiation, thereby exerting an anti-osteoporosis effect. Therefore, QGY targeting CKIP-1 to regulate the AKT/mTOR-autophagy signaling pathway may represent a promising drug candidate for the treatment of osteoporosis.

## INTRODUCTION

Osteoporosis (OP) is a systemic multifactorial skeletal disorder characterized by decreased bone mass, deterioration of bone microarchitecture and increased bone fragility, resulting in a tendency of fracture and leading to possible lifelong disability or death [1, 2]. With the expanding ageing population, the prevalence of OP is increasing, and it has become an important public health concern in many countries [3]. Researches have shown that the imbalance between osteoblast-mediated bone formation and osteoclast-mediated bone resorption, which leads to OP [4]. Among them, the imbalance in the number and activity of osteoblasts and osteoclasts plays a major role [5]. Considering that the adverse reactions of the present used drugs in clinical with accurate efficacy are also obvious, the development of possible novel therapeutic strategies has become of real importance.

CKIP-1 mediates cell growth, differentiation, apoptosis, cytoskeleton [6, 7], and bone formation, and has been found to be a promising target for OP therapy. Studies have shown that CKIP-1 knockout (KO) mice increase bone density and bone mass with age, and the activity of osteoblasts is significantly higher than those of wild-type mice [8], while the small interfering RNA of CKIP (siRNA) significantly promoted bone formation *in vivo* and *in vitro* [9]. Autophagy is the main catabolic process for eukaryotic cells to degrade and recycle damaged macromolecules or organelles. As a cell survival pathway, it plays a vital role in maintaining bone homeostasis, and the changes in this pathway are to some extent associated with osteoporosis [10–13].

Herbal medicines, especially traditional Chinese medicines (TCMs), which are based on natural compounds, have been clinically used in Asian countries for more than 2,500 years, for the treatment of various diseases including osteoporosis [14–17]. For example, Bu-Shen-Ning-Xin decoction was reported to suppress osteoclastogenesis by regulating RANKL/OPG ratio and protected mice from osteoporosis [17]. Another TCM formula, Fufang Zhenshu Tiaozhi (FTZ), also processed anti-osteoporotic activities against aging-induced osteoporosis in mice [14]. Wang et al. reported that another TCM formula Gushukang granules protected mice from osteoporosis by inhibiting osteoclastogenesis and stimulating osteoblastogenesis [16]. Based on the widely published TCM-related studies, few adverse effects were reported. Thus, growing attentions were attracted for the clinical usage and research application of TCMs. QiangGuYin (QGY) is a famous TCM formula that has been widely used in China for the clinical treatment of postmenopausal osteoporosis for almost 20 years [18–20]. In 2017, we published our multi-center clinical trial involving 240 participants. Based on the results of our study, 1-Year treatment with QGY effectively and safely increased Bone Mineral Density (BMD) and reversed osteoporotic bone turn over [20]. The underlying mechanism, however, remained to be uncovered. Therefore, this study tried to explore the potential mechanism of CKIP-1 involved in autophagy in bone cells through the AKT/mTOR signaling pathway and the regulation of QGY.

## MATERIALS AND METHODS

### Experimental animal

Adult female Sprague-Dawley (SD) rats were purchased from the Experimental Animal Center of Zhejiang University of Traditional Chinese Medicine and were fed under environmental conditions without specific pathogens (temperature 20-25° C, relative humidity 55-65%, light/dark cycle 12 h) And maintain free access to water and food. All experimental procedures follow the Guidelines for the Care and Use of NIH Laboratory Animals (NIH Publication No. 80-23, revised in 1978) and have been approved by the Animal Care and Use Committee of Zhejiang University of Traditional Chinese Medicine (Approval Number: 20200622-09).

### Preparation of QGY medicines and pharmacy-containing serums

QGY (No. Z20195155) is produced by the Pharmaceutical Preparation Center of the Second Affiliated Hospital of Zhejiang University of Traditional Chinese Medicine. Each bottle of QGY contains 22.2mL concentrated soup, which were composed of 245g crude drugs, including honeysuckle stem 25 g, Cornu Cervi Degelatinatum 20 g, Caulis Spatholobi 25 g, Nidus Vespae 20 g, *Gentiana macrophylla* 15 g, Radix Sileris 15 g, cinnamon 10 g, *Ligusticum wallichii* 20 g, *Eucommia ulmoides* 15 g, *Astragalus membranaceus* 30g, Rhizoma Drynariae 20 g, and *Dipsacus asperoides* 30 g. The drug-containing serum was prepared as follows: 40 male SD rats weighed 280-300 g were randomly divided into control or drug groups. In drug group, rats were intragastrically administered QGY at a dosage of 10mL/kg twice a day. In control group, PBS was administered instead of QGY. After 3 days, rats were euthanized and blood was isolated by eyeball extirpating. Then, serum from drug group (QGY-serum) and control group (control-serum) were isolated and used *in vitro* experiments.

### Animal osteoporosis model

After 12-week-old female SD rats (body weight 280-300 g) were adaptively fed for 3 days, 5 rats were subjected to a bilateral laparotomy group (Sham), and another 30 rats were subjected to bilateral ovariectomy according to the previous experimental method (OVX) [21]. OVX rats were randomly divided into 6 groups (n=5): vehicle group (OVX+PBS), low-dose QGY group (OVX+L-QGY, 2.5 mL/kg/day), medium-dose QGY group (OVX+M-QGY, 5 mL/kg/day), high-dose QGY group (OVX+H-QGY, 10 mL/kg/day), autophagy inhibitor group (OVX+3-MA), high-dose QGY + autophagy inhibitor group (OVX+H-QGY+3-MA). All drugs were treated orally for 6 weeks. After completing the experiment, samples of the right femur of each group of rats were taken for histological staining detection and micro-CT scan.

### Histomorphometric analysis

A micro-CT imaging system (Skyscan 1176, Belgium) was used for morphological analysis of trabecular bone. The left femur sample was scanned at a resolution of 14.8 μm. Select the trabecular bone region of interest to quantify BMC. By measuring bone trabecular volume fraction (BV/TV, %), bone density (BMC/TV), bone trabecular number (Tb.N) and bone trabecular spacing (Tb.Sp, mm) and other related parameters, Reflects the internal microstructure of the femur.

The femur sample was fixed in 4 % paraformaldehyde, then the sample was embedded in paraffin and cut into 4 μm thick sections. Use hematoxylin-eosin (H&E) staining kit to observe the cell morphology of trabecular bone. In addition, according to the manufacturer’s instructions, slices were fixed in TRAP fixative solution at 4° C for 3 min, then incubated in TRAP working solution at 37° C in the dark for 60 min, stained with hematoxylin solution for 3-5 min, and then subjected to microscopic examination to confirm the bone cell differentiation.

### Bone cell culture and differentiation

MC3T3-E1 osteoblasts and RAW264.7 cells were purchased from Cell Bank of Chinese Academy of Sciences (Shanghai, China). MC3T3-E1 osteoblasts were cultured in α-minimum essential medium (Gibco; Thermo Fisher Scientific, Inc., Waltham, MA, USA) with 10% fetal bovine serum (Gibco) and 1% penicillin-streptomycin. Cells were maintained in medium with ascorbic acid (50 μg/mL) and β-glycerol phosphatase (10 mM) for osteogenic differentiation for 21 days. In addition, RAW264.7 cells were cultured using a-MEM (10% FBS, 1%P/S) and maintained at 37° C with 5% CO_2_ overnight, and were stimulated with M-CSF (20 ng/mL) and RANKL (50 ng/mL) in a-MEM to induce osteoclasts formation (1×10^3^ cells/well in 96-well plates).

### Western blotting analysis

Lyse the tissue or cells in pre-cooled lysate (50 mM Tris-Cl, pH 7.4, 1 mM EDTA pH 8.0, 250 mM NaCl, and 1% Triton-X) for 30 minutes, and centrifuge at 12000 rpm at 4° C for 20 minutes. The supernatant was collected and the total protein concentration was determined by BCA protein analysis kit (Beyotime, Shanghai, China). The same amount of protein (40 μg) was separated by 10% SDS-PAGE and transferred to PVDF membrane (Bio-Rad). The membrane was blocked with 4% skimmed milk for 2 h and incubated overnight at 4° C with the following primary antibodies: CKIP-1 (1:1000, Santa Cruz Biotechnology, SC-376355), LC3II/I (1:1000, Abcam, ab128065), p62 (1:1000, Abcam, ab91526), Atg5 (1:1000, Proteintech, 10181-2-AP), AKT (1:1000, Cell Signaling Technology, 9272), phospho-AKT (1:1000, CST, 4060), mTOR (1:1000, Cell Signaling Technology, 2983), phospho-mTOR (1:1000, Cell Signaling Technology, 2971), RUNX2 (1:1000, Abcam, ab236639), RANKL (1:1000, Santa Cruz Biotechnology, SC-377079), OPG (1:1000, Santa Cruz Biotechnology, SC-390518) and GAPDH (1:5000, Santa Cruz Biotechnology, United States, sc-25778). The membrane was incubated with HRP-conjugated antibody (Wuhan Boster Bioengineering Co., Ltd.) for 1 h, and then the enhanced ECL chemiluminescence reagent (Bio-Rad) and GelDoc imaging system (BioRad) were used to quantify immunoreactive bands.

### Construction of CKIP-1 expression lentivirus and knockdown shRNA

CKIP-1 expression lentivirus and knockdown shRNA were constructed using standard methods. In detail, Taq Plus DNA Polymerase (ET105-02-01, TIANGEN), dNTP (CD117, TIANGEN), T4 DNA Ligase (FL101-02, TRANS), Xho I (1094A, TAKARA), Not I (1166A, TAKARA), DL15000 DNA Maker (MD110, TIANGEN), DL2000 DNA Maker (MD114, TIANGEN) were used according to the manufactures’ instructions. MC3T3-E1 cells and RAW 264.7 cells were then transfected with lentivirus and shRNA using polybrene (Sigma) for 24 h and passaged for following experiments.

### Co-IP detection

Total cellular proteins of osteoblasts from different groups were extracted. Extracted proteins (500 μg/sample) were pretreated with 30 μL Agarose Protein A+G beads (CST, #9863 and 37478) for 2 h at 4° C. Then the beads were removed by centrifuge. After that, 3μg HA-Tag rabbit monoclonal antibody (IP-HA, 1:100, CST, #3724) or IgG (IP-IgG, 1:100, CST, #3900) was added into samples and incubated overnight at 4° C. Another 30μl Agarose Protein A+G beads (50%) were added into samples and incubated for 6h at 4° C. After centrifuge, the sediment was isolated and resuspended with 30μL loading buffer. Then, the proteins were measured by western blot as described above.

### LC3 immunofluorescence assay

The cells were fixed in 4% paraformaldehyde (Solarbio, Beijing, China) and blocked in 5% BSA for 1 h, and then incubated with rabbit monoclonal anti-LC3B antibody (Cell Signaling Technology, Danvers, MA, USA) overnight and with Alexa Fluor The 488-conjugated secondary antibody was incubated for 1h. Finally, it was counterstained with 4',6-dimidyl-2-phenylindole dye (DAPI) (Cell Signaling Technology, Danvers, MA, USA), and performed on a confocal laser scanning microscope (Leica, SP8, Germany) Capture images on.

### ELISA analysis

7 days after osteoblast induction, ELISA was used to measure the protein expression levels of RANKL and OPG according to the manufacturer's instructions.

### Quantitative real-time PCR

Total RNA was removed from the cells using Trizol reagent (Invitrogen, Carlsbad, NM, USA) according to the manufacturer’s instructions. cDNA was synthesized with ReverTra Ace qPCR RT Master Mix (Toyobo, Osaka, Japan). Real-time PCR was performed on an ABI 7500 Fast Real-Time PCR System using SYBR Green PCR Master Mix (Applied Biosystems, Foster City, CA, USA). The optimal conditions for thermal cycling are as follows: initial denaturation at 95° C for 15 min, followed by denaturation at 95° C for 30 s, annealing at 60±3° C for 30 s, and extension at 60° C for 30 s for 40 cycles. The primers used for real-time PCR are as follows: CKIP-I (Forward): 5'-GCCGTGAGTCCTGAAGAGAAG-3', (Reverse): 5'-CGAGTAGGGTGGGCAAGATAG-3'; OPG (Forward): 5'-CCTGCCTGGGAAGAAGATCA-3', (Reverse): 5'-TTGTGAAGCTGTGCAGGAAC-3'; RANKL (Forward): 5'-GCACACCTCACCATCAATGCT-3', (Reverse): 5'-GGTACCAAGAGGACAGAGTGACTTTA-3'; GAPDH (Forward): 5'-ATGGGTGTGAACCACGAGA-3', (Reverse): 5'-CAGGGATGATGTTCTGGGCA-3'.

### Alizarin Red Staining

The cells were fixed with 4% paraformaldehyde (Solarbio, Beijing, China) for 10 min at room temperature. According to the manufacturer's instructions, the cells were stained with Alizarin Red Staining Kit (Beyotime, Shanghai, China) for 60 min at room temperature. After washing 3 times, photograph the stained cells immediately.

### Statistical analysis

All data were presented as mean ± standard deviation (mean ± SD). Differences among data from 3 or more groups were analyzed by one-way ANOVA with post hoc Tukey’s multiple comparison tests. Analyses were performed using Prism software (v. 8.0; GraphPad, San Diego, CA, USA). *p*<0.05 were recognized as statistically significant.

## RESULTS

### QGY improves bone condition in osteoporotic rats

**Figure 1:**
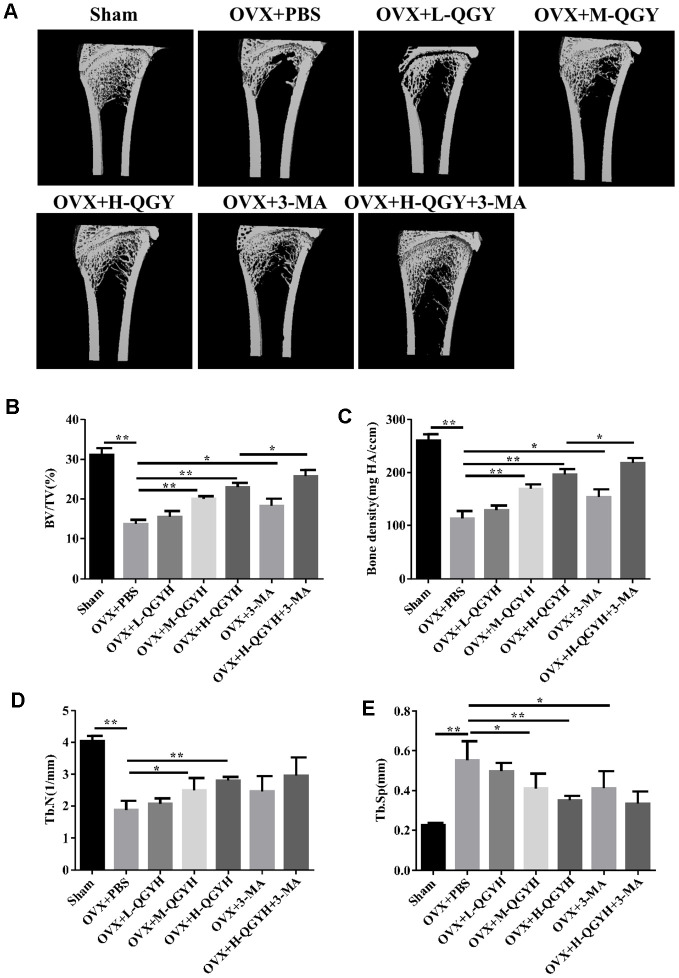
**Micro-CT analysis after treating with QGY in animal model.** Ovariectomy (OVX) was performed in rats, and then different doses of QGY were given to the rats. (**A**) Representative figures of three-dimensional micro-CT analysis. (**B**–**E**) Quantitative analysis of bone trabecular volume fraction (BV/TV, %) (**B**), bone density (BMC/TV) (**C**), bone trabecular number (Tb.N) (**D**) and bone trabecular spacing (Tb.Sp, mm) (**E**) in three-dimensional micro-CT analysis. The data are presented as the means ± SD (n=5). ^*^p<0.05, ^**^p<0.01. One-way ANOVA followed by Tukey's post hoc test.

To determine whether QGY restored bone loss caused by estrogen deficiency, OVX rats were treated with different doses of QGY for 6 weeks. The results of micro-CT three-dimensional imaging showed that compared with the Sham group, the OVX+PBS group had lower bone density and reduced bone trabeculae; QGY and autophagy inhibitor 3-MA treatments could improve bone conditions, and the effective effect of QGY was dose-dependent (Figure 1A). Further analysis of the image parameters found that compared with the Sham group, the BV/TV, BMC/TV, Tb.N index of the OVX+PBS group decreased, and the TB.Sp index increased; QGY treatment significantly increased BV/TV and bone density, and decreased Tb Sp, and is dose-dependent; autophagy inhibitor 3-MA treatment can significantly increase BV/TV and bone density. At the same time, high-dose QGY and 3-MA treatment can significantly improve bone microstructure (Figure 1B).

**Figure 2:**
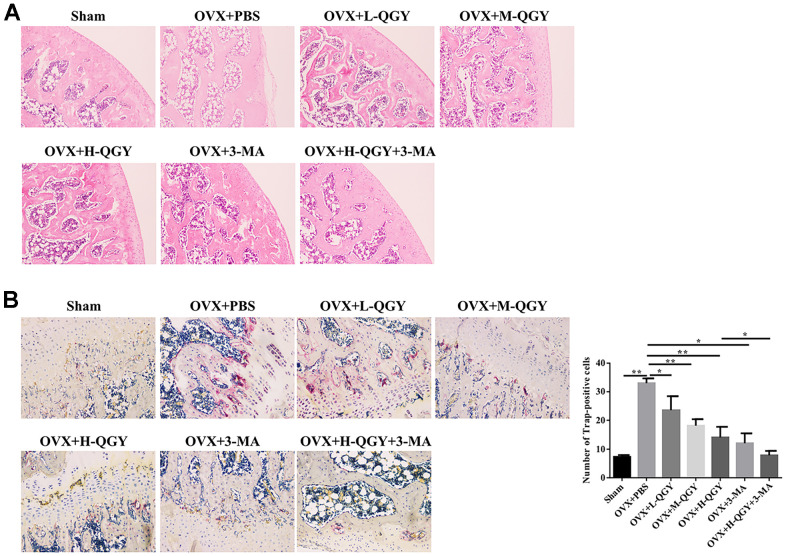
**Staining analysis after treating with QGY in animal model.** (**A**) H&E staining of the trabecular bone. (**B**) TRAP staining and quantitative analysis of the trabecular bone. The data are presented as the means ± SD (n=5). ^*^p<0.05, ^**^p<0.01. One-way ANOVA followed by Tukey's post hoc test.

To further evaluate the influence of QGY on bone structure, H&E staining was used to determine the thickness and number of bone trabeculae. Compared with the Sham group, the bone marrow cavity in the OVX+PBS group became larger, the bone trabeculae became thinner, and the structure was disordered and loose; QGY and 3-MA treatments thickened the bone trabeculae to varying degrees, the bone marrow cavity became smaller, and the structure was compact (Figure 2A). In order to evaluate the effect of QGY on osteoclasts, TRAP staining was used to determine the differentiation of osteoclasts. TRAP staining showed that the osteoclasts were wine-red, located at the edge of the trabecular bone. Compared with the Sham group, the number of osteoclasts in the OVX+PBS group was significantly increased; QGY and 3-MA treatments significantly reduced the number of osteoclasts (Figure 2B). These results indicate that QGY can improve the bone condition of osteoporotic rats in a dose-dependent manner, possibly by regulating autophagy.

### QGY reduces the expression of CKIP-1 in the femoral tissue of osteoporotic rats and mediates autophagy through the AKT/mTOR pathway

**Figure 3:**
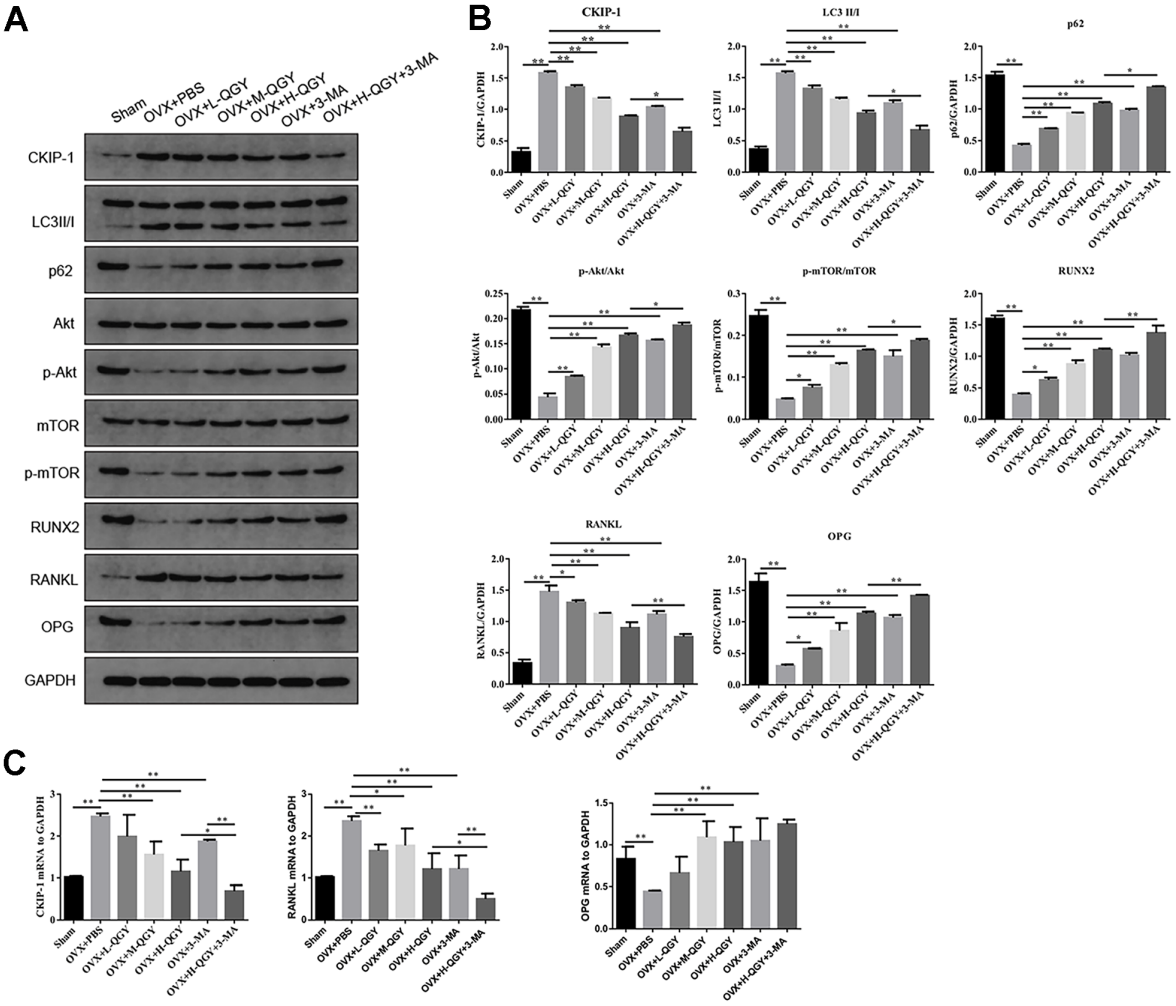
**The effect of QGY on the protein expression of femoral tissue in osteoporotic rats.** (**A, B**) Representative figures (**A**) and quantitative analysis (**B**) of protein expression of CKIP-1, LC3II/I, p62, p-AKT/AKT, p-mTOR/mTOR, RUNX2, RANKL and OPG. (**C**) Gene expression levels of CKIP-1, RANKL and OPG. The data are presented as the means ± SD (n=5). ^*^p<0.05, ^**^p<0.01. One-way ANOVA followed by Tukey's post hoc test.

In order to study the molecular mechanism of QGY improving osteoporosis, the expression of related proteins in the autophagy pathway mediated by CKIP-1 and AKT/mTOR was evaluated. Western blotting analysis showed that, compared with the Sham group, the expressions of CKIP-1, LC3II/I and RANKL were up-regulated in the femoral tissues of the OVX+PBS group, while the expressions of p62, p-AKT, p-mTOR, RUNX2, and OPG were down-regulated, suggesting that the increase of autophagy levels inhibit the AKT/mTOR signaling pathway; QGY and 3-MA treatment reverses the expression level of related proteins, and the effect of QGY shows a dose-dependent characteristic (Figure 3A, 3B). The gene expression levels of CKIP-1, RANKL and OPG were further checked, and it was found that QGY and 3-MA treatments also reduced the gene expression levels of CKIP-1 and RANKL and promoted the gene expression level of OPG (Figure 3C). It is speculated that QGY may inhibit autophagy, activate the AKT/mTOR pathway, reduce the expression of CKIP-1 and RANKL, and promote the expression of RUNX2 and OPG, thereby improving osteoporosis.

### QGY mediates the effect of CKIP-1 on autophagy of osteoblasts and osteoclasts

**Figure 4:**
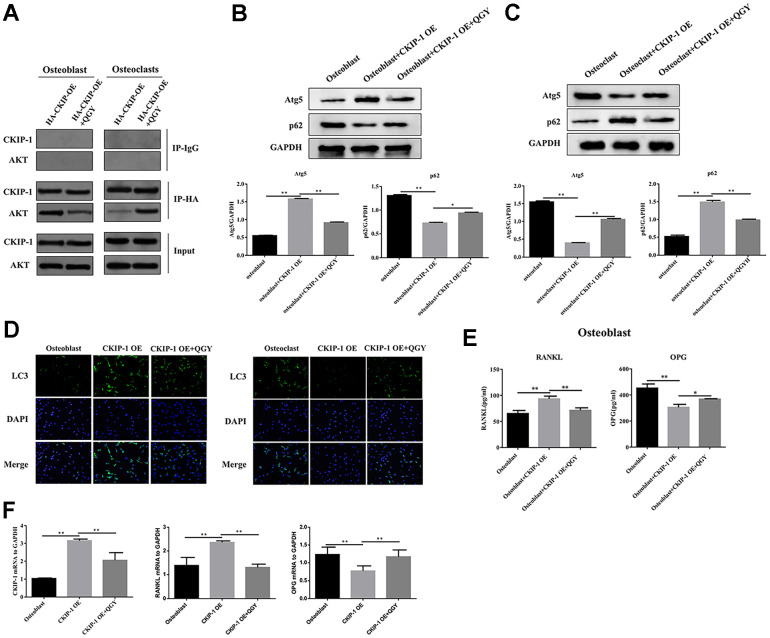
**QGY mediates the effect of CKIP-1 on autophagy through the AKT/mTOR pathway.** (**A**) Co-IP assay in the osteoblasts and osteoclasts. (**B**, **C**) Representative figures and quantitative analysis of protein expression of Atg5 and p62 in the osteoblasts (**B**) and osteoclasts (**C**). (**D**) LC3 immunofluorescence assay in the osteoblasts and osteoclasts. (**E**) RANKL and OPG content analysis in the osteoblasts. (**F**) Gene expression levels of CKIP-1, RANKL and OPG in the osteoblasts. The data are presented as the means ± SD (**B**, **C**, **E**, **F**: n=6; **D**: n=3). ^*^p<0.05, ^**^p<0.01. One-way ANOVA followed by Tukey's post hoc test.

In order to study the mechanism of QGY-mediated CKIP-1 in improving osteoporosis, the interaction between CKIP-1 and AKT was first evaluated. The results showed that in osteoblasts, compared with the HA-CKIP-1-OE group, the binding content of CKIP-1 and AKT decreased significantly after QGY treatment, indicating that CKIP-1 and AKT interact, and QGY treatment can reduce its binding capacity; the opposite trend appears in osteoclasts (Figure 4A). These results indicate that there is an interaction between CKIP-1 and AKT, and QGY inhibits the binding of CKIP-1 and AKT in osteoblasts and promotes the binding of CKIP-1 and AKT in osteoclasts.

**Figure 5:**
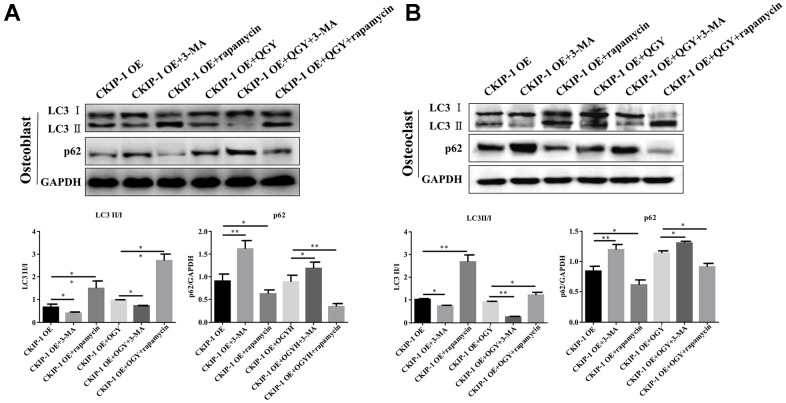
**QGY mediates the effect of CKIP-1 on autophagy of osteoblasts and osteoclasts.** (**A**, **B**) Representative figures and quantitative analysis of protein expression of LC3II/I and p62 in the osteoblasts (**A**) and osteoclasts (**B**). The data are presented as the means ± SD (n=6). ^*^p<0.05, ^**^p<0.01. One-way ANOVA followed by Tukey's post hoc test.

Further analyze the effect of QGY on autophagy. First, it was determined that QGY could reduce the mRNA level after CKIP-1 overexpression (Figure 4F). In osteoblasts, compared with the control group, the Atg5 protein expression level of the CKIP-1 OE group increased, the p62 protein level decreased (Figure 4B), and the LC3 punctate aggregates (autophagosomes) increased (Figure 4D), indicating that CKIP-1 mediates autophagy activation of osteoblasts. On the contrary, in osteoclasts, compared with the control group, the Atg5 protein expression level of the CKIP-1 OE group decreased, the p62 protein level increased (Figure 4C), and the LC3 punctate aggregates decreased (Figure 4D). This indicates that CKIP-1 mediated autophagy inhibition, and QGY treatment reversed CKIP-1 mediated osteoblasts and osteoclast autophagy. Next, the regulation of autophagy was tested. In osteoblasts, compared with the CKIP-1 OE infection group, after the addition of autophagy inhibitor 3-MA, the intracellular LC3 II/I level decreased, and the p62 protein expression level increased; after the addition of the autophagy activator rapamycin, LC3 II/I level increased, but p62 protein expression level decreased; after adding QGY treatment, LC3 II/I level decreased, p62 protein expression level increased; autophagy inhibitor 3-MA treatment can increase the autophagy inhibitory effect of QGY on cell autophagy, and autophagy activator rapamycin can also increase the activation of QGY on cell autophagy (Figure 5A). In osteoclasts, this trend is reversed (Figure 5B). These results indicate that QGY inhibits the combination of CKIP-1 and AKT, activates the AKT/mTOR pathway, inhibits autophagy in osteoblasts, thereby improving osteoporosis, which is consistent with the results of *in vivo* experiments.

### The effect of CKIP-1 mediated autophagy on the differentiation and function of osteoblasts and osteoclasts

**Figure 6:**
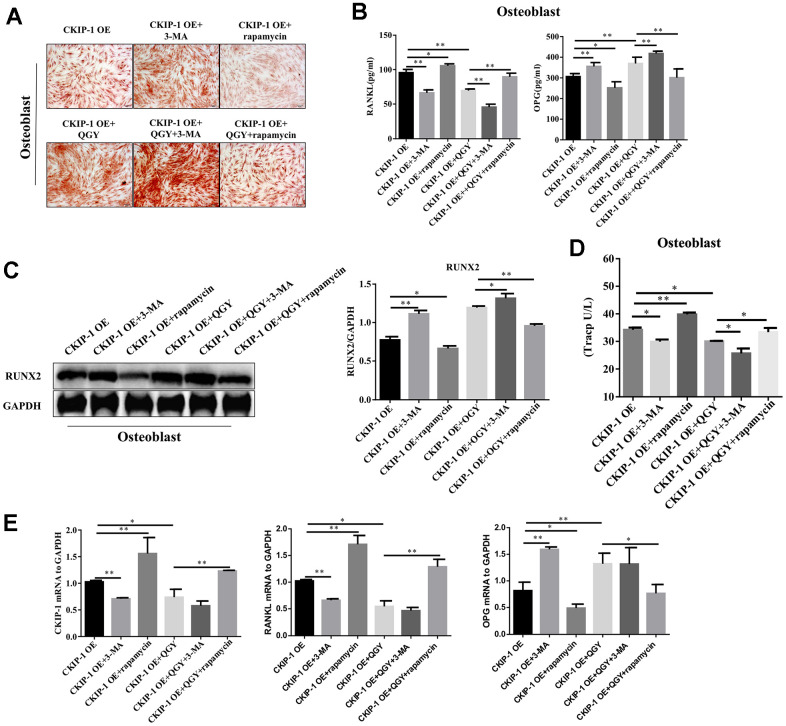
**CKIP-1 mediated autophagy on the differentiation and function.** (**A**) Alizarin Red Staining in the osteoblasts. (**B**) RANKL and OPG content analysis in the osteoblasts. (**C**) Representative figures and quantitative analysis of protein expression of RUNX2 in the osteoblasts. (**D**) TRACP activity analysis in the osteoblasts. (**E**) Gene expression levels of CKIP-1, RANKL and OPG in the osteoblasts. The data are presented as the means ± S.D (n=6). ^*^p<0.05, ^**^p<0.01. One-way ANOVA followed by Tukey's post hoc test.

The differentiation ability of osteoblasts was evaluated by Alizarin Red staining. The results showed that 3-MA treatment can significantly increase the calcified nodules in osteoblasts infected with CKIP-1 OE, and the osteogenic capacity is enhanced; after rapamycin treatment, the calcified nodules are reduced and the osteogenic capacity is reduced; after QGY treatment, the calcified nodules are obvious, and the osteogenic ability is enhanced; 3-MA can enhance the deposition of QGY on the calcified nodules of osteoblasts, and rapamycin can inhibit the promotion of QGY on the calcified nodules of osteoblasts (Figure 6A). These results indicate that QGY promotes the differentiation ability of osteoblasts and enhances the osteogenic ability by inhibiting the level of autophagy.

Further detect the content of RANKL and OPG in the supernatant of osteoblasts. The results showed that compared with the control group, the RANKL content in the supernatant of CKIP-1 OE group was significantly increased, and the OPG content was significantly reduced; QGY treatment could reduce RANKL content and increased OPG content, indicating that QGY inhibits osteoblast secretion of RANKL and promotes OPG secretion (Figure 4E). Treatment with autophagy inhibitor 3-MA can significantly reduce RANKL secretion and increase OPG secretion in osteoblast, and increase the regulation of QGY on RANKL and OPG secretion; treatment with autophagy inducer rapamycin can increase RANKL secretes and inhibits OPG secretion, and inhibits the regulation of QGY (Figure 6B). Similarly, the mRNA levels of RANKL and OPG showed a consistent trend (Figures 4F, 6E). These results indicate that QGY inhibits the secretion of RANKL by osteoblasts and promotes the secretion of OPG by regulating autophagy, which is consistent with the results of *in vivo* experiments.

Western blotting analysis was used to detect the expression level of osteoblast marker RUNX2 protein. The results show that the autophagy inhibitor 3-MA can promote the protein expression of RUNX2 in the osteoblasts CKIP-1 OE group; the autophagy activator rapamycin can inhibit the protein expression of RUNX2; QGY treatment can increase the protein expression of RUNX2. In addition, 3-MA can enhance the promotion of QGY on RUNX2 expression, while rapamycin can block the induction of QGY on RUNX2 expression in osteoblast (Figure 6C). To evaluate the anti-tartrate acid phosphatase (TRACP) activity of osteoclasts. The results showed that treatment with 3-MA can inhibit TRACP activity of osteoclasts infected by CKIP-1 OE; treatment with rapamycin can increase TRACP activity. In addition, QGY treatment can inhibit TRACP activity; 3-MA can enhance the inhibitory effect of QGY on osteoclast TRACP activity, and rapamycin can inhibit the inhibitory effect of QGY (Figure 6D). These results indicate that QGY inhibits TRACP activity through autophagy regulation on osteoclast.

## DISCUSSION

**Figure 7:**
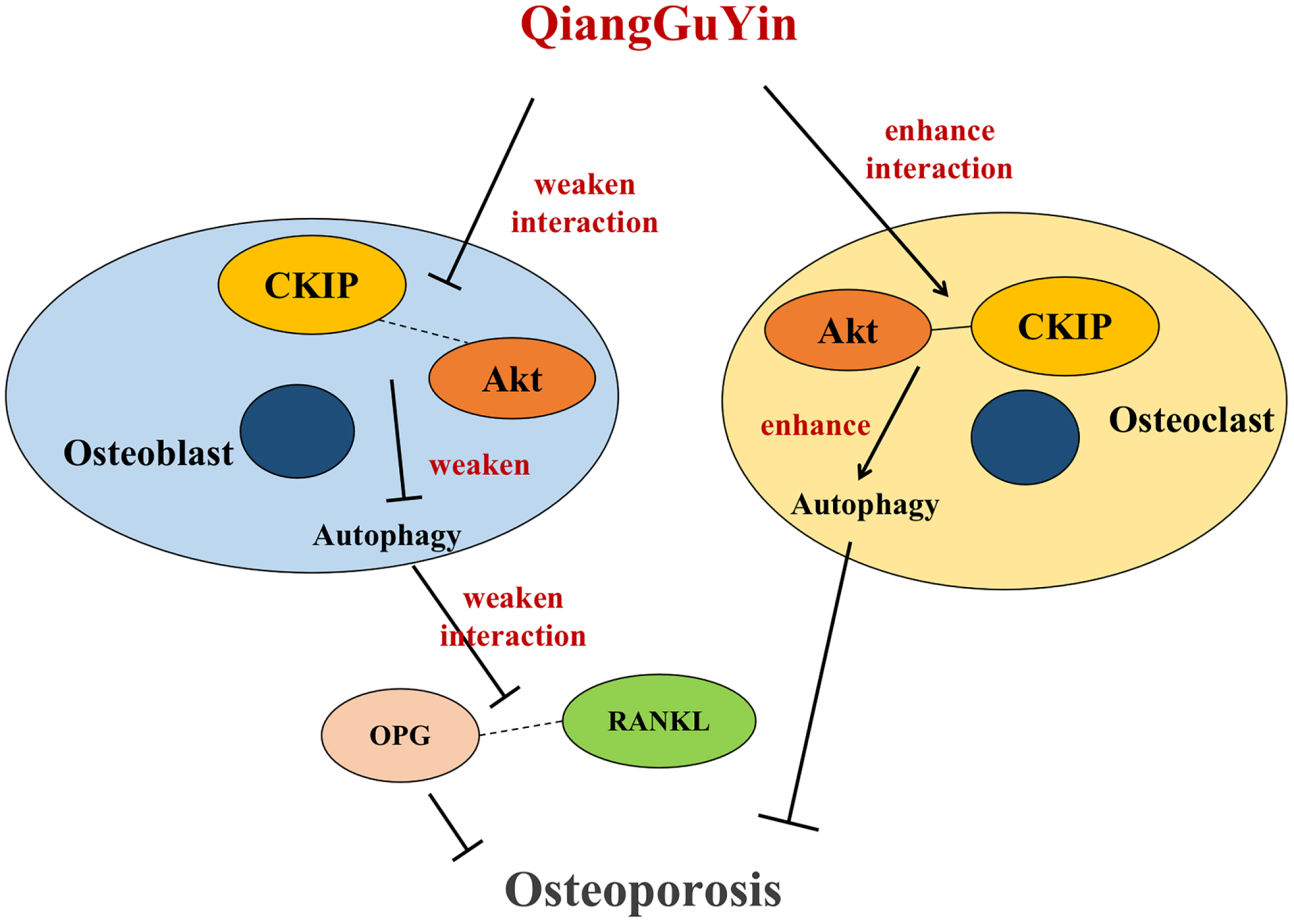
Schematic diagram of QGY differential regulation of CKIP-1/AKT/mTOR/autophagy pathways in osteoblasts and osteoclasts.

CKIP-1 is a transcription factor that acts as a scaffold linker to mediate interactions with various signals and cellular proteins, and is considered to be an important gene responsible for the development and progression of osteoporosis. Previous studies have shown that CKIP-1 is a regulator of cell viability, apoptosis, cytoskeleton formation and cell differentiation. Recent studies have also confirmed that CKIP-1 is a negative regulator of bone formation, leading to excessive activation of osteoclasts and bone loss. Drugs targeting osteoblasts CKIP-1 can reverse bone formation and prevent osteoporosis [22]. In CKIP-1 KO mice, osteoblast activity and BMD were significantly increased [23]. SiRNA knockout targeting CKIP-1 improved bone formation and osseointegration in a rat model of osteoporosis [24]. In another study using a rat mandibular distraction osteogenesis (DO) model, CKIP-1 silencing inhibited BMSC apoptosis and promoted osteogenic differentiation [25]. Consistent with previous studies, our research shows that CKIP-1 mediates the autophagy activation of osteoblasts and inhibits differentiation, increases the expression ratio of RANKL/OPG in osteoblasts, and then inhibits cell osteogenesis; CKIP-1 also mediates the autophagy inhibition of osteoclast. Importantly, QGY treatment promotes osteoblast differentiation and enhances osteogenic capacity by targeting CKIP-1 mediated AKT/mTOR pathway activation and autophagy inhibition (rather than osteoclasts), thereby playing a role in improving osteoporosis (Figure 7).

The AKT/mTOR pathway is a key regulator of bone formation [26]. Previous studies have shown that AKT knockout mice have delayed ossification [27]. AKT is also a key factor in the differentiation process of osteoblasts [28]. Xi et al. reported that the AKT/mTOR signaling pathway participates in the inhibition of osteoporosis by promoting osteoblast proliferation, differentiation and bone formation *in vitro* and *in vivo* models [29]. In this study, we found that overexpression of CKIP-1 significantly inhibits the AKT/mTOR pathway, while OGY treatment reactivates the inhibited AKT/mTOR pathway, which is consistent with previous studies [9, 30–35]. Therefore, CKIP-1 acts as an intermediary between QGY and AKT/mTOR signaling crosstalk. After treating osteoblasts with AKT/mTOR pathway inhibitors, it is further proved that QGY exerts anti-osteoporosis effects through AKT/mTOR pathway. In addition, the AKT/mTOR signaling pathway is also one of the most important ways to regulate autophagy. The autophagy pathway is considered to play a supporting role in the development of osteoporosis [36–39]. In this study, autophagy was activated during osteoblast differentiation. Among them, ATG5 and p62 have been shown to regulate autophagy degradation [40], and the lipidated form of LC3 converted from LC3-I to LC3-II is considered as an autophagosome marker because it locates and aggregates on autophagosomes [41]. QGY treatment targets CKIP-1 mediated AKT/mTOR pathway activation and autophagy inhibition.

RANKL (receptor activator of nuclear factor kappa B ligand) is a key osteoclast differentiation factor, which is secreted in large quantities by fibroblasts, osteoblasts and stromal cells around the prosthesis. It combines RANK and cooperates with NF- κB, promote osteoclast differentiation and survival, and osteoclast bone resorption; OPG, as an osteoclast differentiation inhibitor, usually competes with RANK to integrate RANKL, blocking the promotion effect of RANKL/RANK on osteoclast and osteoclast bone resorption, thereby inhibiting osteolysis [42]. The imbalance between osteoblast bone formation and osteoclast bone resorption ultimately leads to osteolysis around the prosthesis, where the OPG/RANKL ratio is one of the best characterizing biomarkers related to the pathology of bone destruction [43]. As expected, our results indicate that QGY treatment also promotes osteoblast differentiation and enhances osteogenic capacity, thereby playing a role in improving osteoporosis.

Our data is also supported by other reports. For example, NUPR1 is considered to be a new type of bone formation regulator, and its deficiency will reduce the production of early osteoclasts and enhance the production of osteoblasts, resulting in increased bone formation [44]. In addition, resveratrol regulates the expression of autophagy-related proteins in a dose-dependent manner, such as p62, LC3-II, Atg5, Atg7 and Atg12, etc., inhibits autophagy in osteoblasts and activates osteoclast autophagy, and regulates autophagy. It promotes osteoblast differentiation and inhibits osteoclast differentiation, thereby improving postmenopausal osteoporosis [45]. Consistently, our study also showed the opposite autophagy expression and osteogenic ability in osteoblasts and osteoclasts, which may be related to the restoration of the balance between bone formation and bone resorption by QGY regulating the AKT/mTOR signaling pathway mediated by CKIP-1.

To sum up, consistent with our previously published clinical trial [20], this study provides new information that QGY treatment promotes growth by targeting CKIP-1 mediated AKT/mTOR pathway activation and autophagy inhibition. Osteocytes differentiate and enhance the ability of bone formation, thereby playing a role in improving osteoporosis. Importantly, activation of autophagy with rapamycin not only reversed the inhibition of QGY on the RANKL/OPG ratio and osteoclast differentiation, but also further enhanced this inhibition by using 3-MA to inhibit autophagy. The synergistic *in vivo* anti-osteoporosis effect of QGY and 3-MA confirms the results we found in cell research. As far as we know, the current research reveals for the first time the CKIP-1 regulatory mechanism of this popular herbal formula to treat osteoporosis.
